# Pursuing Quality Education in Physical and Rehabilitation Medicine in Japan

**DOI:** 10.3389/fresc.2022.877986

**Published:** 2022-07-22

**Authors:** Toyoko Asami

**Affiliations:** Department of Rehabilitation Medicine, Saga University Hospital, Saga, Japan

**Keywords:** quality, education, Physical and Rehabilitation Medicine, motivation, curiosity, digital transformation

## Abstract

In Japan, medical education and training are the combined responsibility of two ministries namely Ministry of Education, Culture, Sports, Science and Technology, and the Ministry of Health, Labor and Welfare. The medical education system underwent a major transformation in August 2021 making it a seamless clinical education blending pre-graduation and post-graduation training. Not all universities offer rehabilitation medicine curriculum. Furthermore, where rehabilitation medicine is taught, the curriculum content is not standardized. All medical students sit for a common national medical practitioner qualifying examination. However, only a few questions on Rehabilitation Medicine are included. The personal experience of the author's teachings in rehabilitation medicine at Saga University medical school is described. Emphasis is placed on experiential learning on subjects that are current and state-of-the-art in Japan including robotics. It is aimed at promoting inspired motivation for the students to pursue specialized training in rehabilitation medicine. Japan can take lessons from the European Union's white book on Physical Medicine and Rehabilitation as well as the International Society of Physical and Rehabilitation Medicine core curriculum. In addition, the Rehabilitation Medicine education system can be further improved through a well-coordinated preclinical and clinical medical education. There is also a need to expand the rehabilitation medicine field and address the gaps with other specialties.

## Introduction

The objective of this paper is to describe the Japanese medical school education system highlighting the rehabilitation medicine component at undergraduate and postgraduate levels.

## Current Medical Education System

**Figure 1:**
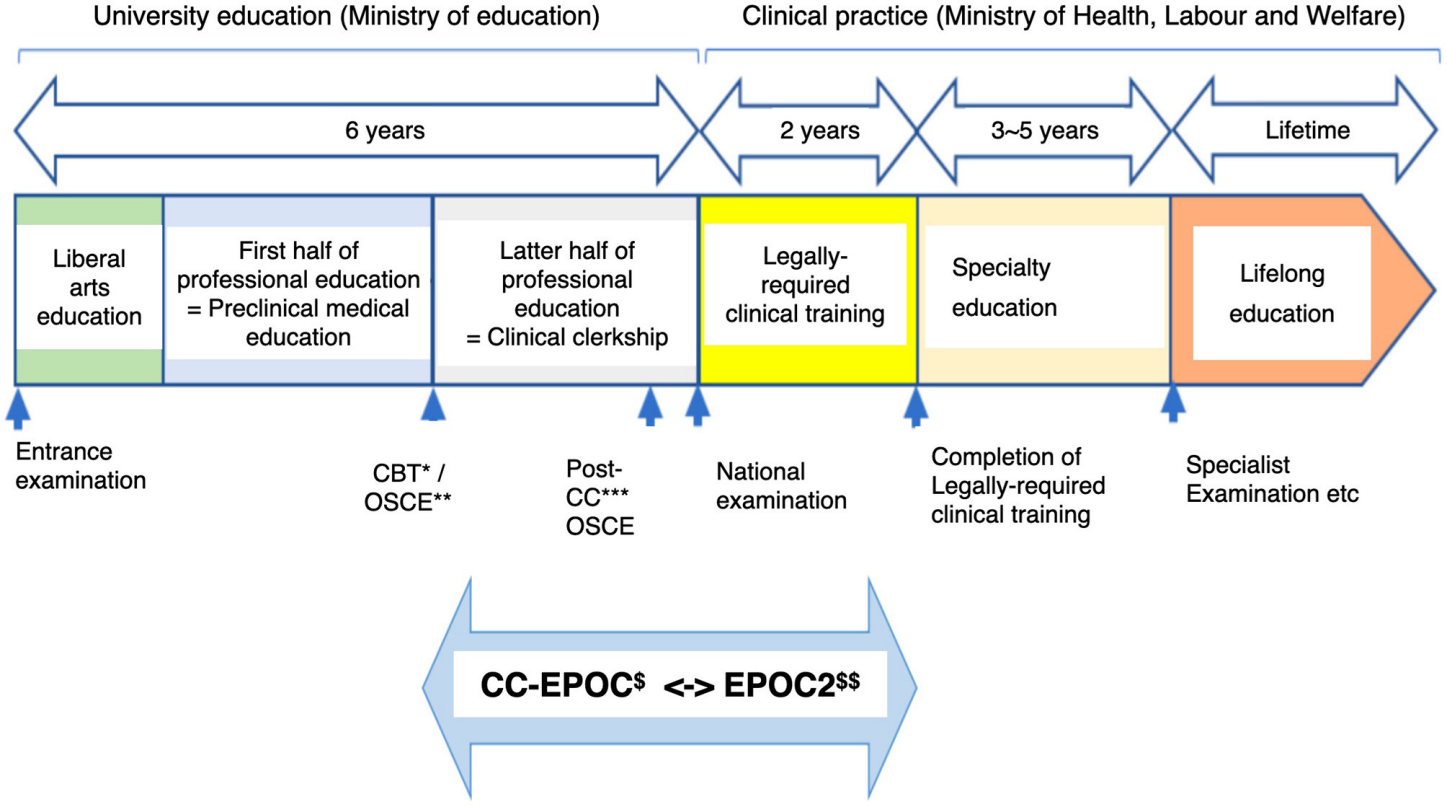
Seamless clinical education before and after graduation of medical school in Japan since August 2021. *Computer based testing. **Objective structured clinical examination. ***Post-clinical clerkship. ^*$*^Clinical clerkship-evaluation system of postgraduate clinical training. ^*$$*^E-portfolio of clinical training.

Since its early beginnings in 2021, medical education has undergone transformation toward meeting the needs of the Japanese population. Table 1 shows the changes in the medical school education system since 1991 (1). In August 2021, a landmark development took the form of a seamless clinical education for under and post graduate medical students as shown in Figure 1 (2).

**Table 1:** Medical education in Japan before 2021.

**Before 1945 (end of World War II)**	**4-year education (2-year basics, 2-year clinical medicine lectures, doctor's license is automatically granted)**
1945	Started efforts to improve pre-graduate medical education
1946	Resumed national examination for doctors
1948	Internship system started
1968	Clinical training system implementation 1 year
2004	Two-years clinical training under the law (formal internship)
	The doctor training course has a total of 8 years, 6 years before graduation and 2 years after graduation training.
2016	Medical education model core curriculum started
2020	Plan to start of clinical training that integrates qualifications and ability requirements in pre- and post-graduation

*Based on reference (1)*.

It has been common practice that high school graduates can pursue medical studies which conventionally consists of 6 years of medical education governed by the Ministry of Education, Culture, Sports, Science and Technology (MEXT). An internship of 2 years follows, and this is under the governance of Ministry of Health, Labor, and Welfare. Specialty education (residency) is then continued under the latter ministry and lasts for 3–5 years depending on the specialty. For Rehabilitation Medicine, the residency training is 3 years. All doctors are then expected to continue with lifelong education.

The August 2021(Figure 1) revised seamless clinical education blends pre-graduation and post-graduation training aimed at standardizing the expansion of the medical school education framework. Pre-graduation medical education consists of preclinical medical education and clinical clerkship. Preclinical medical education is provided by education specialists using a schedule standardized for school education. Clinical clerkship is conducted in a community practice likened to an apprenticeship.

Up till now, many medical schools throughout Japan have offer rehabilitation medicine courses, but the curriculum content is not standardized. Some offer up to 20 h, while others just offer a few hours of rehabilitation medicine teaching.

Numerous universities offer only a few days of rehabilitation clinical training in clinical clerkship. Furthermore, the National Medical Practitioners Qualifying Examination has included only a few questions related to rehabilitation medicine.

Only 2 to 3% of interns learn about rehabilitation medicine through their respective postings. This indicates that most of them are not exposed to rehabilitation medicine, hence very few interns venture into that field. Those who intend to make rehabilitation medicine their specialty have to learn the majority of their practice during the 3 years of residency. But there are far too few instructors in that sector of medical education.

## Current Needs for Rehabilitation in Medical Service

Rehabilitation medicine is a unique field that focuses on activities. It needs to meet the major demands placed upon it in recent years by Japan's aging society. The average lifespan in 2020 was 81.64 years for males and 87.74 years for females^1^, making Japan one of the countries with the longest life expectancy in the world. In 2020 the aging rate was 28.8%^2^. Generally speaking, one's healthy lifespan is approximately 10 years shorter than one's overall life expectancy, and as a result an increased aging rate is directly related to an increase in the number of individuals living with disabilities. With the advances in acute medical care increasing survival rates, residual polymorbidity and decreased reserve capacity in the elderly lead to increased numbers of old people aging with disabilities. This results in the increase in demand for rehabilitation services.

When one examines trends in occupations in rehabilitation in Japan, one notes that physical therapy and occupational therapy came under national licensing status in 1965. Speech-language-hearing therapists came under national licensing status in 1997. These professionals took responsibility for highly specialized forms of rehabilitative medical care for children and disaster victims with disabilities. The acceleration in the number of older persons in the 2000s resulted in the increase of the total number of therapists by 8.7 times, from 32,400 in 1998 to 282,400 in 2018.The specialization system for physiatrists (rehabilitation physicians) started in 1980 as one of 18 clinical specialties. Although the number of physiatrists showed an upward trend, in 2019 there were just 2,531, i.e., only 1.3% of specialists in all fields ^2^.

With regards to healthcare services covered by the medical insurance system, rehabilitation fees account for only 5% of all medical costs (3, 4). In the midst of social conditions in which there is a major effort to reduce total medical costs, the clear expansion of rehabilitation medicine is causing stress amongst stakeholders.

## Personal Experience at Saga University Medical School

The author has been passionate about and has dedicated herself to medical education, clinical practice, and research at both universities and university hospitals since her graduation in 1984. Education is the cornerstone of the sustainable development of rehabilitation medicine and standards (3). For the training program in rehabilitation medicine have been set. At Saga University, this is as shown in Table 2 (5).

**Table 2:** Duration of rehabilitation medicine training for medical students and residents at Saga University.

**Grade**	**Time**
1st grade of medical school	6 h (visit)
4th grade of medical school	6 h (lecture)
5th grade of medical school	Selective training
6th grade of medical school	Selective training
Resident 2nd grade	Selective training

Japanese rehabilitation medicine education demands that students learn a great deal, and exemplary services and research have emerged amongst the crème de la crème of the specialty. At Saga University there are only two rehabilitation medicine instructors for both the pre- and post-graduate levels. Medical School classes are small. The two are also responsible for engaging approximately 3,000 patients per year. In addition, they are responsible for handling approximately 28,000 physical therapy, 10,000 occupational therapy and 7,000 speech-language-hearing therapy cases per year.

**Figure 2:**
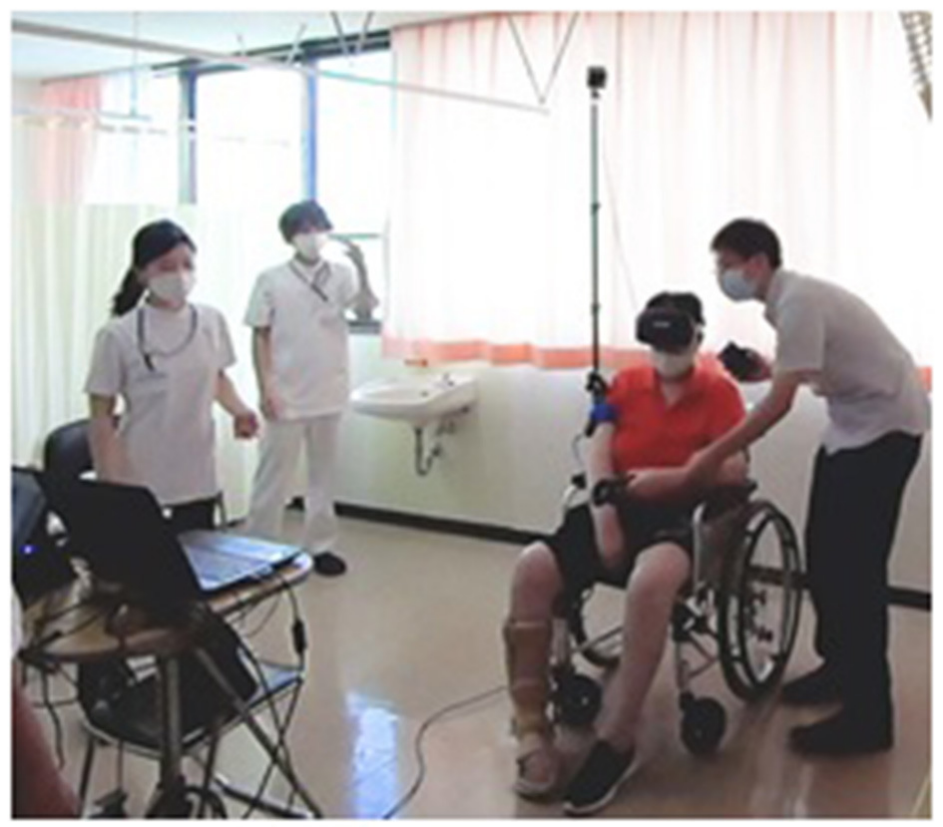
mediVR KAGURA^®^ (VR, virtual reality).

In spite of lack of academic resources and overwhelming workload in clinical practice, research, and education, the author has attempted to provide “handmade” medical education. One is through clinical education with the use of rehabilitation robotics, including virtual reality (VR) and myoelectric prosthetic hands. These are among the most advanced medical devices that can stimulate the curiosity of young people (Figure 2) (6). At Saga University Hospital, Japan's first “Robot Rehabilitation Outpatient Care” was launched on October 1, 2014. Its purpose was to create a high-quality rehabilitation healthcare system that utilizes rehabilitation robotics. Patients are referred from all over Japan, which ultimately contributes a critical mass for an education system (7, 8). This initiative utilizes participatory clinical training for medical students, interns, and residents. It is an education system that allows independent participation making it easier for students to comprehend what is being taught. Experience in rehabilitation medicine deepens making it useful for solving problems using artificial intelligence (AI). Advances in education that utilize these kinds of digital transformation (DX) methods have accelerated during the COVID-19 pandemic. They can in fact be used to supplement actual, in-person training which had been restricted by the pandemic.

## Discussion

The August 2021 landmark initiative taken by two distinct Ministries responsible for churning out medical specialists is promising. It brings Japan closer to the current practice in Europe that evolved out of the sheer need for standardization for the European Union where medical positions among countries is seamless. Japan's Ministry of Education, Culture, Sports, Science and Technology (MEXT) along with its partner Ministry of Health, Labor, and Welfare can take lessons from this (?) years of collaborative work amongst member countries The European Union's white book on Physical Medicine and Rehabilitation and the International Society of Physical and Rehabilitation Medicine (ISPRM) core curriculum can provide an excellent insight to an educational and clinical framework (refs?). However, it is necessary to keep in mind that clinical practice is not a pure science and that it is a system that comprises many kinds of wisdom, including skills that have been developed over a long history of tinkering. Thus, clinical education should not be just school education or just apprenticeship, but rather it needs to be both.

Motivation is an important element in educational success (9). Curiosity, the bedrock of internal motivation for learning, along with inspiration and external motivation can be provided through apprenticeship that can make up for the aspects of the school education model that are lacking. When considering these issues, one realizes that the education system should not be constructed in such a way as to encourage overcrowding but rather should have slack in certain circumstances. The creation of a flexible education system that takes advantage of the unique features of rehabilitation medicine, while at the same time maintaining a sufficient grasp of medical education as a whole, is likely to be the key to the future of rehabilitation medicine. Importance should be placed on learning clinical expertise with a focus on actual practice and on promoting “inspired motivation,” which is a type of external motivation found, for example, in apprenticeship. In addition, through the adoption of the new concept of “activity” into medicine, curiosity, which is a form of internal motivation, will be further emphasized. The author believes that there is a need to emphasize this within the limitations of the undergraduate education system. Strengthening the rehabilitation medicine studies at medical school level serves as a sustainable feeder to a continuous birth of rehabilitation physicians who can serve the ever-increasing needs of an aging population such as in Japan. Only then will the World Health Organization's (WHO) realization of meeting the rehabilitative needs of the 2.4 billion world population be met (ref). Japan is particularly challenged by gaps in numbers of high schoolers, medical students and interns who are yet to be exposed to rehabilitation medicine through their education experience. Though the numbers of rehabilitation physicians are far too small to meet the demands of student teachings, the combined effort with rehabilitation inter-,trans- and multi-disciplinary teams through a structured rehabilitation medicine curriculum can be key in tackling this problem.

## Conclusion

Highlighting the rehabilitation medicine component at undergraduate and postgraduate levels of the Japanese medical school education system has revealed the shortcomings of clinical education in Japanese medical education. In order to solve this problem advances must be made in coordinating pre-clinical medical education and clinical clerkship. In the field of rehabilitation medicine education, there is a need to solve specific problems related to coordination with pre-clinical medical education while keeping in mind effective clinical clerkship education. In the meantime, there is a need to expand the field and address the gap compared to other specialties. What is necessary when doing this is not to disregard the inspired motivation of apprenticeship that promotes sufficient motivation and curiosity.

## Data Availability Statement

The raw data supporting the conclusions of this article will be made available by the authors, without undue reservation.

## Author Contributions

The author confirms being the sole contributor of this work and has approved it for publication.

## Conflict of Interest

The author declares that the research was conducted in the absence of any commercial or financial relationships that could be construed as a potential conflict of interest.

## Publisher's Note

All claims expressed in this article are solely those of the authors and do not necessarily represent those of their affiliated organizations, or those of the publisher, the editors and the reviewers. Any product that may be evaluated in this article, or claim that may be made by its manufacturer, is not guaranteed or endorsed by the publisher.

## Footnotes

^1^Available online at: https://www.mhlw.go.jp/toukei/oshirase/.

^2^Available online at: https://www8.cao.go.jp/kourei/whitepaper/w-2021/zenbun/03pdf_index.html.

## References

[1] Fukushima O. History of medical education in Japan. Med Educ. (2018) 49:421–8.

[2] National University Hospital Council of Japan. The World's First Seamless Evaluation System for Medical Education From Medical Students to Residents at the National Level (in Japanese). Available online at: https://www.umin.ac.jp/publications/press-release/20210315cc-epoc-detailed.pdf

[3] e-Stat:Statistics of Japan- Statistics of Medical Care Activities in Public Health Insurance. Available online at: https://www.e-stat.go.jp/stat-search/files?page=1&toukei=00450048&tstat=000001029602

[4] e-Stat :Statistics of Japan -Statistics of Long-term Care Benefit Expenditures. Available online at: https://www.e-stat.go.jp/en/statistics/00450049

[5] *Reiwa 4th Year Curriculum Guidelines (in Japanese)*. Saga University School of Medicine, Department of Medicine.

[6] Hara M Kitamura T Murakawa Y Shimba K Yamaguchi S Tamaki M. Safety and feasibility of dual-task rehabilitation program for body trunk balance using virtual reality and three-dimensional tracking technologies. Prog Rehabil Med. (2018) 3:20180016. 10.2490/prm.20180016 32789241 PMC7365202

[7] Asami T. Points related to the introduction and utilization in myoelectric prostheses. Jpn J Rehabiil Med. (2018) 57:227–33. 10.2490/jjrmc.55.227

[8] Asami T Kitajima M Nanri Y Murata K Sato T. Case report on long-term, continuous improvement of walking ability as a result of botulinum toxin injection therapy and low-frequency rehabilitation with HAL. Int J Phys Med Rehabil. (2016) 4:1000339.

[9] Norman DA. Things That Make Us Smart. New York, NY: Addison-Wesley Publishing Co (1993).

